# Transcatheter aortic valve replacement in failed transcatheter versus failed surgical bioprostheses: a systematic review with exploratory meta-analysis

**DOI:** 10.3389/fcvm.2026.1870804

**Published:** 2026-09-18

**Authors:** Mohammad Ghannam, Mustafa Abomohsen, Iyad Y. Idries, Fayez Shamoon, Muhammad Ghallab, Fawzi Zghyer, Alena V. González, Abdullah Ahmad, Habib A. Habib, Rahul Vasudev

**Affiliations:** 1 Brookdale University Hospital and Medical Center, Brooklyn, NY, United States; 2 St. Joseph’s University Medical Center, Paterson, NJ, United States; 3 Department of Cardiovascular Medicine, Creighton University, Omaha, NE, United States; 4 Heart, Vascular and Thoracic Institute, Cleveland Clinic Foundation, Cleveland, OH, United States; 5 Department of Cardiology, Heart Failure Division, University of Alabama at Birmingham, Birmingham, AL, United States

**Keywords:** redo TAVR, systematic review, TAV-in-SAV, TAV-in-TAV, TAVR

## Abstract

**Background:**

With the expanding use of transcatheter aortic valve replacement (TAVR) in younger patients, the need for reintervention on failed bioprostheses is rising. Comparative evidence between redo TAVR for failed transcatheter valves (TAV-in-TAV) and TAVR for failed surgical valves (TAV-in-SAV) is limited.

**Objective:**

To systematically review and meta-analyse the clinical, haemodynamic, and safety outcomes of TAV-in-TAV versus TAV-in-SAV.

**Methods:**

PubMed, Scopus, Web of Science, Cochrane CENTRAL, and Embase were searched from inception to April 2026. Studies directly comparing TAV-in-TAV with TAV-in-SAV and reporting original data with ≥10 patients per group were included. Risk of bias was assessed using ROBINS-I and the Newcastle-Ottawa Scale. Random-effects meta-analyses were performed for 30-day mortality, 1-year mortality, mean transvalvular gradient at 30 days, and new permanent pacemaker (PPM) implantation.

**Results:**

Four observational studies, including 705 patients in the extracted comparative cohorts, met the inclusion criteria. Two full-text studies contributed to the primary quantitative analyses. TAV-in-TAV was not associated with a statistically significant difference in 30-day mortality compared with TAV-in-SAV (OR 0.71, 95% CI 0.25–2.00), nor in 1-year mortality (OR 1.02, 95% CI 0.47–2.18). TAV-in-TAV was associated with lower mean transvalvular gradients at 30 days (MD −2.71 mmHg, 95% CI −4.07 to −1.34). A non-significant trend toward higher new permanent pacemaker implantation was observed after TAV-in-TAV (OR 1.85, 95% CI 0.94–3.65).

**Conclusion:**

Current comparative evidence between TAV-in-TAV and TAV-in-SAV remains limited and observational. No definitive mortality difference was identified, but clinically meaningful benefit or harm cannot be excluded. TAV-in-TAV shows a consistent early haemodynamic signal with lower gradients, while the possible increase in pacemaker implantation remains exploratory. These findings should be considered hypothesis-generating and require confirmation in larger prospective studies with longer follow-up.

**Systematic Review Registration:**

https://www.crd.york.ac.uk/PROSPERO/view/CRD420261369410, PROSPERO CRD420261369410.

## 1 Introduction

Transcatheter aortic valve replacement (TAVR) has fundamentally transformed the management of severe aortic stenosis. Initially developed for patients at prohibitive or high surgical risk, TAVR has progressively expanded to intermediate- and low-risk populations, with accumulating evidence supporting its use even in younger patients (1, 2). As a result, the number of TAVR procedures performed worldwide has increased substantially over the past decade, accompanied by improvements in device technology and procedural outcomes (3).

With the expansion of TAVR into lower-risk and longer-living populations, the long-term durability of bioprosthetic valves has become an increasingly important clinical concern. Structural valve degeneration (SVD) remains an inherent limitation of all bioprosthetic valves, with surgical bioprostheses demonstrating rates of degeneration of approximately 5%–10% at 10 years (4). Although long-term data for transcatheter heart valves are still evolving, similar degenerative processes are anticipated, raising the likelihood of an increasing need for reintervention in the coming years (5).

For patients with failed surgical aortic bioprostheses, valve-in-valve TAVR (TAV-in-SAV) is an established treatment strategy that provides a less invasive alternative to redo surgery, with favourable short-term outcomes and acceptable mid-term survival in high-risk populations (6, 7). In contrast, redo TAVR for failed transcatheter valves (TAV-in-TAV) represents an emerging therapeutic approach, for which the current evidence base remains limited. Most available data derive from retrospective cohorts and registry-based studies with heterogeneous patient populations, variable follow-up durations, and inconsistent outcome definitions (8, 9).

Direct comparative evidence between TAV-in-TAV and TAV-in-SAV is scarce, and no randomised controlled trials have been conducted to date. Existing observational studies suggest potential differences in procedural success, haemodynamic performance, and complication profiles; however, findings remain inconsistent and are limited by methodological heterogeneity and small sample sizes (9–11). Consequently, the relative benefits and risks of these two reintervention strategies are not yet clearly defined.

Given the growing population of patients undergoing TAVR and the anticipated rise in bioprosthetic valve failure, there is a critical need to synthesise the available evidence to inform clinical decision-making. Therefore, the present systematic review and exploratory meta-analysis aims to evaluate and compare the clinical, haemodynamic, and safety outcomes of TAV-in-TAV versus TAV-in-SAV based on currently available comparative studies.

## 2 Methods

This study was conducted following the Cochrane Handbook for Systematic Reviews of Interventions (12). The results were reported as specified by the Preferred Reporting Items for Systematic Reviews and Meta-Analyses (PRISMA) statement (13). Additionally, this study has been registered with PROSPERO under the identification number (CRD420261369410).

### 2.1 Information sources and search strategy

A comprehensive electronic search was conducted in PubMed/MEDLINE, Embase, Scopus, Web of Science Core Collection, and Cochrane CENTRAL from inception to April 2026. The search combined controlled vocabulary and free-text terms related to transcatheter aortic valve replacement, redo or repeat TAVR, valve-in-valve procedures, failed transcatheter valves, and failed surgical bioprostheses. The full database-specific search strategies are provided in Supplementary Table A1. No language or publication-date restrictions were applied. Two reviewers independently performed the searches and screened the retrieved records. Duplicates were removed before title and abstract screening.

### 2.2 Eligibility criteria

- Inclusion Criteria
  - **Population**: Adult patients (≥18 years) with a degenerated or failed bioprosthetic aortic valve (either transcatheter or surgical) undergoing reintervention.
  - **Intervention**: Valve-in-valve transcatheter aortic valve replacement (TAV-in-TAV) using any approved transcatheter heart valve system.
  - **Comparator**: Valve-in-valve TAVR into a failed surgical aortic bioprosthesis (TAV-in-SAV).
  - **Outcomes**: Studies reporting at least one of the following outcomes were included: all-cause mortality (30-day, 1-year, or longest available), procedural success (VARC-2/3 criteria), mean transvalvular gradient (mmHg), aortic valve area (cm^2^), aortic regurgitation (≥moderate or mild), new permanent pacemaker implantation, stroke, major bleeding, acute kidney injury, coronary obstruction, hospital length of stay, or early safety composite endpoints.
- Exclusion Criteria

Studies were excluded if they lacked a direct comparison between **TAV-in-TAV** and **TAV-in-SAV**, did not provide original extractable outcome data, or had fewer than **10 patients per group**. Reviews, editorials, commentaries, letters, cross-sectional studies, animal studies, *in vitro* studies, and duplicate or overlapping cohorts were excluded, with only the most complete report retained when overlap existed. Studies were also excluded when the failed valve type could not be clearly identified. Immediate bailout TAV-in-TAV procedures performed for acute index-procedure complications were excluded from the primary quantitative analysis unless separate data were available for delayed/elective redo TAVR. Conference abstracts were considered only for narrative synthesis when sufficient numerical data were available and were not pooled if reporting was limited or the population differed substantially from the target redo-valve-failure population.

#### 2.2.1 Screening and data management

The screening process was carried out in two distinct phases. The first phase involved the initial screening of titles and abstracts to identify potentially relevant studies. In the second phase, full-text articles of selected studies were thoroughly reviewed to confirm eligibility. Rayyan software was used to facilitate the screening process. Two independent reviewers conducted the eligibility assessment, and any disagreements were resolved by consulting a third reviewer (14).

### 2.3 Data extraction

A standardised data extraction form was developed in Microsoft Excel and piloted on three randomly selected studies before use. Two reviewers independently extracted data from each included study; discrepancies were resolved by discussion or by consulting a third reviewer. From each study, we extracted study characteristics (author, year, design, country), patient baseline demographics (age, sex, STS score, comorbidities), procedural details (access route, valve type, predilation, postdilatation), haemodynamic outcomes (mean and peak gradients, aortic valve area, aortic regurgitation, left ventricular ejection fraction), and clinical safety outcomes (all-cause mortality at 30 days, 1 year and 3 years; stroke; major bleeding; acute kidney injury; new permanent pacemaker implantation; hospital length of stay; and early safety composite endpoints).

For studies reporting continuous data as median and interquartile range (IQR), the mean and standard deviation were estimated using the method of Wan et al. (mean = median, SD = IQR/1.35). When necessary, study authors were contacted for additional or unpublished data, but no responses were received. Extracted data were entered into R (version 4.4.1) for subsequent meta-analysis.

### 2.4 Risk of bias assessment

Non-randomized studies were assessed using the Risk Of Bias In Non-randomized Studies of Interventions tool (ROBINS-I). The assessed domains included bias due to confounding, bias in the selection of participants, bias in the classification of interventions, bias due to deviations from intended interventions, bias due to missing data, bias in the measurement of outcomes, and bias in the selection of the reported result. Overall judgments were categorized as low, moderate, serious, critical risk of bias, or no information (15).

### 2.5 Publication bias

As indicated by Egger et al., the assessment of potential publication bias within this review utilizing Egger’s test for funnel plot asymmetry is not feasible when the number of included studies is fewer than ten (16).

### 2.6 Choice of the meta-analysis model

Given the small number of eligible comparative studies, the primary approach was a structured narrative synthesis of study characteristics, patient populations, procedural details, haemodynamic outcomes, and clinical safety outcomes. Exploratory quantitative pooling was performed only for outcomes reported by at least two studies with sufficiently comparable definitions and time points. Both common-effect and random-effects models were generated for transparency. However, because each pooled analysis included only two studies, the pooled estimates were considered descriptive and hypothesis-generating. Between-study variance and I^2^ estimates were interpreted cautiously because these measures are unstable and poorly informative when *k* = 2.

### 2.7 Calculation of missing data & heterogeneity

In instances where data were reported as the median and interquartile ranges (IQR), conversions were performed to obtain mean (M) and standard deviation (SD) utilizing the equations established by Wan et al. (17). Furthermore, when the standard deviation was not supplied, it was derived from the standard error using the formula for a single sample: SD = SE*√n, where (n) denotes the sample size (18). If the mean change (MC) between baseline and endpoint data was unavailable, it was calculated from the pre-treatment and post-treatment means using the equation MC = Mpost-treatment - Mpre-treatment. Additionally, in cases where the standard deviation of the mean change was not provided, it was computed from the standard deviations of the pre-treatment and post-treatment samples using the formula SD = √(SD^2^pre-treatment+SD^2^post-treatment) (19). Effect sizes (d) and effect size correlations (r) that were missing were calculated through the following formulas: d = (Mtreatment—Mcontrol)/SDpooled, with SDpooled calculated as √[(SD^2^treatment + SD^2^control)/2] (20), and r = d/√(d^2^ + 4) (21).

Heterogeneity was assessed visually using forest plots and statistically using Cochran’s *Q* test and the I^2^ statistic. Because all primary pooled outcomes included only two studies, heterogeneity estimates were interpreted cautiously. Sensitivity analyses were planned when clinically or methodologically appropriate.

## 3 Results

### 3.1 Search and screening

The database search identified a total of 4,920 records from five electronic databases: PubMed/MEDLINE (*n* = 1,187), Scopus (*n* = 451), Web of Science (*n* = 938), Embase (*n* = 2,266), and Cochrane Library/CENTRAL (*n* = 78). After removal of 1,607 duplicate records, 3,313 unique records remained for title and abstract screening. During title and abstract screening, 2,898 records were excluded because they were not relevant to the review question. Therefore, 415 reports were initially sought for retrieval. Of these, 358 reports were not retrieved, leaving 57 full-text reports for eligibility assessment.

After full-text assessment, 53 reports were excluded for not meeting the eligibility criteria. The most common reasons for exclusion included irrelevant population, absence of direct comparison between TAV-in-TAV and TAV-in-SAV, non-original articles, insufficient extractable outcome data, or overlapping/non-eligible study populations. Finally, 4 studies (9–11, 22) met the inclusion criteria and were included in the systematic review. Of these, 2 studies provided sufficiently comparable quantitative data for the primary meta-analyses after exclusion of studies unsuitable for pooling because of methodological or clinical heterogeneity. The study selection process is summarized in Figure 1.

**Figure 1:**
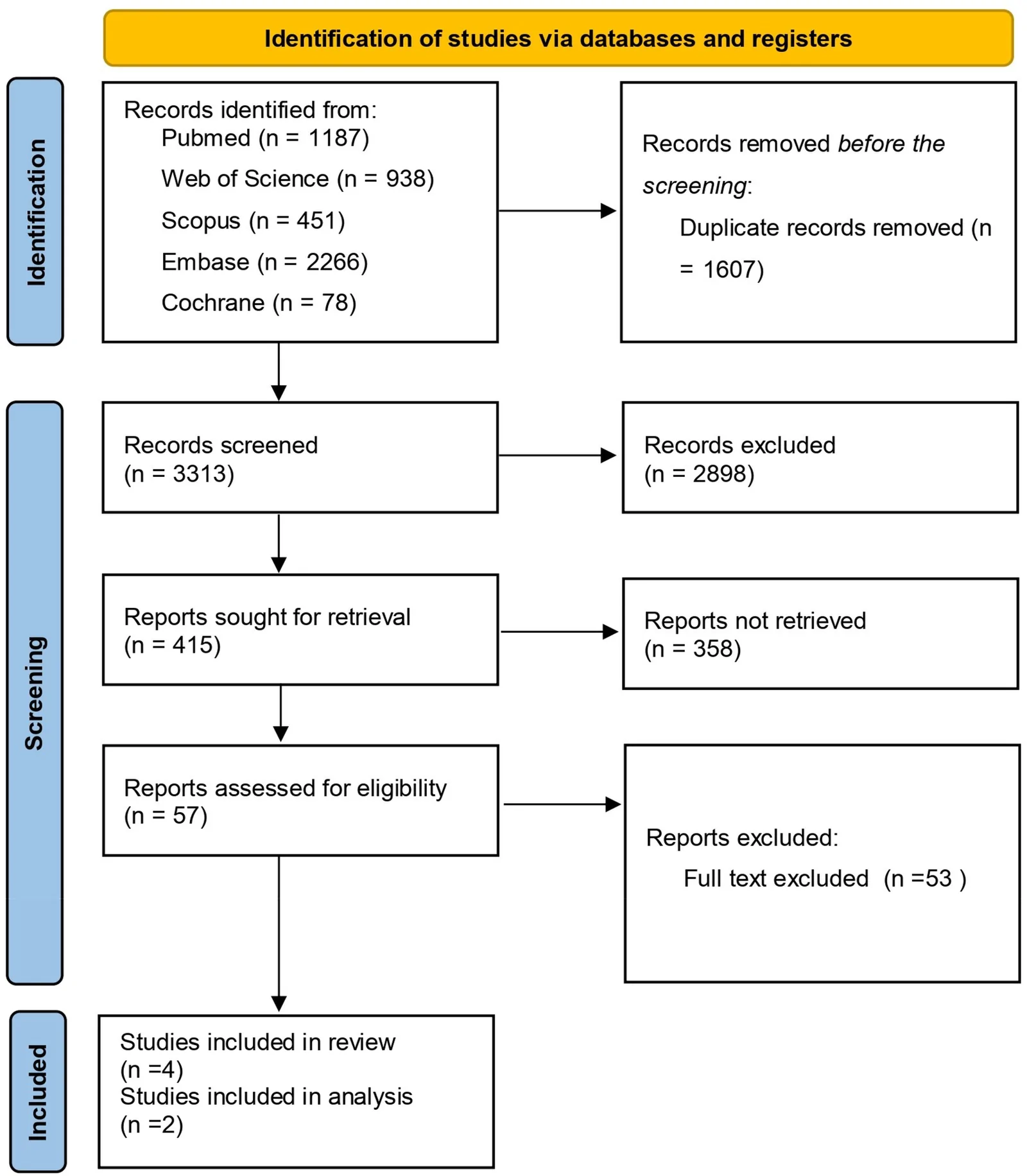
PRISMA flow chart for study screening and inclusion.

### 3.2 Study characteristics

The four included studies were published or available between 2019 and 2026. All were observational in design: Raschpichle et al. (11) was a retrospective cohort study, Rosenzveig et al. (10) was a retrospective TriNetX propensity-matched cohort study, Goppel et al. (22) was an abstract-only retrospective analysis of prospectively collected data, and Landes et al. (9) was an international registry-based propensity-matched cohort analysis. A total of 705 patients were analysed across the four studies, with individual sample sizes ranging from 99 to 330 (matched pairs). The number of TAV-in-TAV patients ranged from 25 to 165, and TAV-in-SAV patients from 74 to 165.

Key baseline characteristics are summarised in Table 1. Patients undergoing TAV-in-TAV were generally older (by 3–6 years) and had a higher prevalence of chronic obstructive pulmonary disease and chronic kidney disease, although these differences were not always statistically significant. The time from index valve implantation to redo procedure was consistently shorter for TAV-in-TAV (median 3.0–3.2 years) compared to TAV-in-SAV (median 9–10 years). Indications for redo varied: most TAV-in-SAV patients presented with aortic stenosis (59%–77%), while a substantial proportion of TAV-in-TAV patients in the Goppel study (90%) underwent redo for intraoperative paravalvular regurgitation rather than structural degeneration. This fundamental difference in indication (PVL vs. stenosis) represents a critical limitation of the Goppel study, as it compares two clinically distinct scenarios. Therefore, it was retained for narrative discussion but excluded from the primary quantitative synthesis.

**Table 1:** Study and patient baseline characteristics.

Characteristic	Raschpichle et al. (11)	Landes et al. (9), matched cohort	Rosenzveig et al. (10), matched cohort	Goppel et al. (22)
Country/centers	USA+Germany, 3 centers	37 centers, Europe/North America/Middle East	USA, TriNetX federated database	Germany, single center
Study period	Nov 2009—May 2018	Apr 2005—Apr 2019	Jan 2010—Jan 2022	Oct 2007—Jul 2017
Study design	Retrospective cohort	International registry-based propensity-matched retrospective cohort	Retrospective TriNetX propensity-matched cohort	ESC abstract: retrospective analysis of prospectively collected database
Sample size used in the review	99	330 matched patients	170 matched patients	106
TAV-in-TAV, *n*	25	165	85	31
TAV-in-SAV, *n*	74	165	85	75
Age, years	83.0 (80.0–86.0)/77.0 (69.2–86.0)	80 (75–84)/79.72 (74–84)	Matched exact age NR; pre-match 74.18 ± 8.47/69.70 ± 10.56	78 ± 8/75 ± 8
Female sex, %	60.0/50.0	40.0/39.4*	Matched NR; pre-match 39.47/35.21	35/40
STS score, %	7.44 (5.65–14.2)/6.80 (3.74–12.3)	6.83 (4.8–9.5)/6.5 (4.8–9.6)	NR	4.6 ± 2.8/5.2 ± 4.0
NYHA III/IV, %	96.0/97.3	77.6/78.2	NR	NR
LVEF, %	59.0 (55.0–69.0)/60.0 (44.0–65.0)	55 (45–60)/55 (45–60)	NR	Baseline NR; discharge 49 ± 16/49 ± 12
Diabetes, %	44.0/24.7	27.3/29.7	NR	NR
COPD/severe pulmonary disease, %	60.0/27.0	24.2/23.6	Matched NR; pre-match chronic lower respiratory disease 58.77/41.90	NR
CKD/renal dysfunction	CrCl <60: 60.0/56.2	eGFR: 49 (35–60)/50 (32–67) mL/min	Matched NR; pre-match CKD 57.90/38.03	NR
Prior CABG, %	28.0/41.9	26.1/27.9	NR	NR
Prior PCI, %	20.0/10.8	NR	NR	NR
Atrial fibrillation/flutter, %	44.0/35.1	28.5/29.7	NR	NR
Time from the index valve to redo	3.2/10 years	3.0 (0–5)/9.0 (6–12) years	NR	10% of TAV-in-TAV had degeneration after 2614 ± 862 days
Indication: AS, %	56.0/59.5	34.5/35.8	NR	TAV-in-SAV stenosis 77%; TAV-in-TAV mainly PVL 90%
Indication: AR, %	16.0/10.8	35.2/34.5	NR	NR
Indication: mixed AS/AR, %	28.0/29.7	29.7/29.1	NR	NR
PVL indication	PVL cases excluded	Not separately reported in the matched table	NR	TAV-in-TAV: 90% intraoperative PVL
Small index valve, %	0/23.0	27/25	NR	NR

Baseline characteristics of the included comparative studies evaluating TAV-in-TAV versus TAV-in-SAV. Data are presented as mean ± SD, median (IQR), or percentage, according to the reporting format of the original study. Values are shown as TAV-in-TAV/TAV-in-SAV unless otherwise stated. For propensity-matched studies, matched cohort data are presented where available. Rosenzveig et al. (10) reported limited baseline characteristics after matching; therefore, pre-matching values are shown only when matched values were not available. Goppel et al. (22) was available only as a conference abstract and is included for descriptive/narrative purposes.

Procedural characteristics are shown in Table 2. Transfemoral access was the predominant route in all studies (68%–90%). Self-expanding second-generation valves were used in 40%–58% of TAV-in-TAV procedures and 23%–61% of TAV-in-SAV procedures. Procedural success (VARC-2/3) was numerically higher for TAV-in-TAV in both Raschpichler (79.2% vs. 67.6%) and Landes (72.7% vs. 62.4%), but only the latter reached statistical significance (*p* = 0.045).

**Table 2:** Procedural characteristics.

Field	Raschpichle et al. (11)		Landes et al. (9)		Rosenzveig 2025		Goppel et al. (22)	
	TAV-in-TAV	TAV-in-SAV	TAV-in-TAV	TAV-in-SAV	TAV-in-TAV	TAV-in-SAV	TAV-in-TAV	TAV-in-SAV
Access route—transfemoral (%)	83.30%	90.50%	86.10%	87.90%	NR	NR	87%	68%
Second valve type—balloon-expandable (%)	60%	77%	42.4%^a^	39.4%^a^	NR	NR	43%	39%
Second valve type—self-expanding (%)	40%	23%	57.60%	60.60%	NR	NR	57%	61%
Second valve size (mm)	26 (26–26)	23 (23–26)	NR	NR	NR	NR	NR	NR
Predilation performed (%)	40%	1.35%	NR	NR	NR	NR	NR	NR
Postdilatation performed (%)	33.30%	24.30%	NR	NR	NR	NR	NR	NR
Procedural success (VARC-2/3) (%)	79.20%	67.60%	72.70%	62.40%	NR	NR	NR	NR
Conversion to open surgery (%)	0%	0%	0%	1.80%	NR	NR	NR	NR
Coronary obstruction (%)	0%	0%	1.20%	4.20%	NR	NR	NR	NR
Need for second valve (%)	0%	2.70%	NR	NR	NR	NR	NR	NR

Procedural characteristics of TAV-in-TAV and TAV-in-SAV procedures across the included studies. Data are reported as percentages or median (IQR), according to the original publications. Values are shown as TAV-in-TAV/TAV-in-SAV. Procedural or device success definitions followed the criteria used in each original study, most commonly VARC-2/VARC-3 definitions. Rosenzveig et al. (10) did not report detailed procedural characteristics. Goppel et al. (22) was available only as an abstract, and procedural details were limited.

a Calculated as 100% minus self-expanding percentage.

Hemodynamic outcomes are presented in Table 3. At 30 days, the mean transvalvular gradient was significantly lower in TAV-in-TAV compared to TAV-in-SAV in both Raschpichler (12.5 vs. 16.4 mmHg, *p* = 0.043) and Landes (12.6 vs. 14.9 mmHg, *p* = 0.011). Similarly, the aortic valve area was larger in TAV-in-TAV (Landes: 1.55 vs. 1.37 cm^2^, *p* = 0.040). Goppel reported lower mean gradients at discharge for TAV-in-TAV (12 vs. 19 mmHg, *p* < 0.001) and at 12 months (11 vs. 18 mmHg, *p* < 0.001). Mild aortic regurgitation was more frequent after TAV-in-TAV in the Landes study (36.1% vs. 17.2% at 30 days, *p* = 0.003), while moderate or severe regurgitation did not differ significantly between groups.

**Table 3:** Haemodynamic outcomes after TAV-in-TAV versus TAV-in-SAV.

Outcome	Time point	Raschpichle et al. (11)	Landes et al. (9)	Goppel et al. (22)	Rosenzveig et al. (10)
Mean transvalvular gradient, mmHg	Baseline	40.0 (26.0–49.5)/40.0 (26.2–47.8); *p* = 0.939	33 (13–47)/30 (18–45); *p* = 0.591	NR	NR
Mean transvalvular gradient, mmHg	Discharge/day 1	15.0 (11.8–17.5)/16.0 (11.0–21.0); *p* = 0.370	NR	12 ± 5/19 ± 8; *p* < 0.001	NR
Mean transvalvular gradient, mmHg	30 days	12.5 (8.75–16.2)/16.4 (13.0–20.2); *p* = 0.043	12.6 ± 5.2/14.9 ± 5.2; *p* = 0.011	NR	NR
Mean transvalvular gradient, mmHg	1 year	NR	Difference remained statistically significant; exact values NR	11 ± 4/18 ± 9; *p* < 0.001	NR
Peak transvalvular gradient, mmHg	Baseline	63.0 (46.0–105.0)/66.0 (45.0–84.0); *p* = 0.938	NR	NR	NR
Peak transvalvular gradient, mmHg	Discharge/day 1	26.5 (19.8–36.2)/29.0 (20.5–38.0); *p* = 0.249	NR	23 ± 8/33 ± 14; *p* < 0.001	NR
Peak transvalvular gradient, mmHg	30 days	24.0 (17.2–29.8)/29.5 (23.8–36.0); *p* = 0.059	NR	NR	NR
Peak transvalvular gradient, mmHg	1 year	NR	NR	21 ± 8/31 ± 14; *p* < 0.001	NR
Aortic valve area, cm^2^	Discharge/day 1	NR	NR	1.61 ± 0.35/1.18 ± 0.32; *p* < 0.001	NR
Aortic valve area, cm^2^	30 days	NR	1.55 ± 0.5/1.37 ± 0.5; *p* = 0.040	NR	NR
Aortic valve area, cm^2^	1 year	NR	NR	1.63 ± 0.43/1.21 ± 0.36; *p* < 0.001	NR
PVL > mild/≥moderate AR, %	Discharge/day 1	0/2.78; *p* = 1.000	NR	NR	NR
PVL > mild/≥moderate AR, %	30 days	0/3.77; *p* = 1.000	8.4/4.8; *p* = 0.462	NR	NR
Mild AR, %	30 days	NR	36.1/17.2; *p* = 0.003	NR	NR
Mild AR, %	1 year	NR	36.2/12.1; *p* = 0.001	NR	NR
AR grade, 0–3 scale	Discharge/day 1	NR	NR	0.8 ± 0.7/0.4 ± 0.5; *p* = 0.003	NR
AR grade, 0–3 scale	1 year	NR	NR	0.8 ± 0.6/0.4 ± 0.5; *p* = 0.001	NR
Severe patient-prosthesis mismatch, %	Any time	12.5/32.7; *p* = 0.201	NR	NR	NR
LVEF, %	Baseline	59.0 (55.0–69.0)/60.0 (44.0–65.0); *p* = 0.354	55 (45–60)/55 (45–60); *p* = 0.418	NR	NR
LVEF, %	Discharge/day 1	60.0 (50.0–65.0)/60.0 (47.0–65.0); *p* = 0.631	NR	49 ± 16/49 ± 12; *p* = 0.970	NR
LVEF, %	30 days	59.0 (51.0–65.5)/61.0 (54.0–66.0); *p* = 0.810	NR	NR	NR
LVEF, %	1 year	NR	NR	50 ± 10/54 ± 12; *p* = 0.081	NR

Data are presented as mean ± SD, median (IQR), or percentage, according to the reporting format of the original study. Values are shown as TAV-in-TAV/TAV-in-SAV. Landes et al. (9) and Raschpichle et al. (11) provided the 30-day mean transvalvular gradient data used in the primary quantitative synthesis. Goppel et al. (22) reported discharge and 1-year haemodynamic outcomes only and was excluded from the primary pooled gradient analysis because it was available only as a conference abstract and included a clinically different TAV-in-TAV population. Rosenzveig et al. (10) did not report haemodynamic outcomes. Goppel et al. (22) reported aortic regurgitation as AR grade on a 0–3 scale rather than as the percentage of patients with ≥ moderate AR. Therefore, Goppel AR data should not be pooled with categorical ≥ moderate AR outcomes.

Clinical safety and mortality outcomes are summarised in Table 4. Thirty-day mortality was low in both groups across all studies (0%–5.4%), with no statistically significant difference. One-year mortality was 9.4% vs. 13.4% in Raschpichler (log-rank *p* = 0.38) and 11.9% vs. 10.2% in Landes (*p* = 0.633). The only study reporting 3-year outcomes (Rosenzveig) found no difference in all-cause mortality (HR 0.695, 95% CI 0.375–1.287, *p* = 0.24). New permanent pacemaker implantation rates were numerically higher in TAV-in-TAV (16.7% and 10.9%) compared to TAV-in-SAV (5.4% and 7.8%), but the differences were not statistically significant (*p* = 0.1 and *p* = 0.251, respectively). Major bleeding, acute kidney injury, and early safety composite endpoints were similar between groups.

**Table 4:** Clinical safety and mortality outcomes.

Outcome	Time point	Raschpichle et al. (11)			Landes et al. (9)			Rosenzveig 2025			Goppel et al. (22)		
		TAV-in-TAVI (n/N, %)	TAVI-in-SAVR (n/N, %)	p-value	TAV-in-TAVI (n/N, %)	TAVI-in-SAVR (n/N, %)	p-value	TAV-in-TAVI (n/N, %)	TAVI-in-SAVR (n/N, %)	p-value	TAV-in-TAVI (n/N, %)	TAVI-in-SAVR (n/N, %)	*p*-value
All-cause mortality	30-day	1 (4.0%)	4 (5.4%)	1	5 (3.0%)	7 (4.4%)	0.57	NR	NR	NR	0%	0%	NR
	1-year	2 (9.4%)^a^	10 (13.4%)^a^	0.38	12 (11.9%)	10 (10.2%)	0.633	NR	NR	NR	NR	NR	NR
	3-year (if available)	NR	NR	NR	NR	NR	NR	25 (29.4%)	17 (20.0%)	0.24 (HR)	NR	NR	NR
Stroke (any)	30-day	NR	NR	NR	NR	NR	NR	NR	NR	NR	NR	NR	NR
	1-year	NR	NR	NR	NR	NR	NR	NR	NR	NR	NR	NR	NR
Major bleeding (VARC-2/3)	30-day	NR	NR	NR	17 (10.3%)	8 (5.2%)	0.061	NR	NR	NR	NR	NR	NR
Acute kidney injury (stage 2/3)	30-day	NR	NR	NR	7 (4.2%)	2 (1.3%)	0.091	NR	NR	NR	NR	NR	NR
New permanent pacemaker implantation	30-day	4 (16.7%)^b^	4 (5.4%)^b^	0.1	18 (10.9%)	12 (7.8%)	0.251	NR	NR	NR	NR	NR	NR
Major vascular complication	30-day	0%	0%	NR	NR	NR	NR	NR	NR	NR	NR	NR	NR
Myocardial infarction	30-day	NR	NR	NR	NR	NR	NR	part of MACE‡	part of MACE	NR	NR	NR	NR
Endocarditis	Any time	NR	NR	NR	NR	NR	NR	11 (12.9%)	19 (22.4%)	0.06 (HR)	NR	NR	NR
Hospital length of stay (days)	–	9 (4–16)	3 (2–6)	<0.001	NR	NR	NR	NR	NR	NR	NR	NR	NR
Early safety (VARC-2/3 composite)	30-day	23 (92%)	68 (91.9%)	1	116 (70.3%)	119 (72.1%)	0.715	NR	NR	NR	NR	NR	NR

Clinical safety and mortality outcomes reported across the included studies. Values are shown as TAV-in-TAV/TAV-in-SAV and are presented as event number/total number with percentage, percentage only, or hazard ratio, according to the original study. Thirty-day mortality, 1-year mortality, and new permanent pacemaker implantation were included in the primary quantitative synthesis when at least two comparable studies were available. Rosenzveig et al. (10) reported 3-year outcomes only and was summarized narratively.

a Raschpichler: 1-year mortality estimated from KM curves (9.4% of 25 = 2; 13.4% of 74 = 10).

b Pacemaker for high-degree AV block only (not including arrhythmia composite). Rosenzveig: MACE (stroke, cardiac arrest, MI) at 3 years: 26/85 (30.6%) vs. 23/85 (27.1%), HR 0.888, *p* = 0.68.

**Table 5:** Risk of bias assessment using ROBINS-I.

Study	Confounding	Selection	Classification of interventions	Deviations from intended interventions	Missing data	Outcome measurement	Selection of reported results	Overall ROBINS-I judgment
Raschpichle et al. (11)	Moderate: unmatched cohort and baseline imbalance, especially age/COPD	Low	Low	Low	Low to moderate	Low: VARC-2 definitions used	Low	Moderate
Landes et al. (9)	Low to moderate: propensity matching with standardized differences <0.1, but residual confounding remains possible	Low	Low	Low	Low	Low: registry/site-reported VARC-2 outcomes	Low	Low to moderate
Rosenzveig et al. (10)	Moderate: propensity matching on 27 covariates, but residual confounding is likely	Low	Moderate: ICD/procedure-code identification	Low	Low: complete 3-year follow-up reported	Moderate to serious: ICD-based outcomes without core adjudication	Low	Moderate
Goppel et al. (22)	Serious: unmatched groups and fundamentally different indications	Moderate	Moderate	Low	Moderate: 93% follow-up	Moderate: abstract-only, limited methodology	Moderate	Serious

Risk of bias assessment of included non-randomized studies using the ROBINS-I tool. Each study was assessed across the standard ROBINS-I domains: confounding, selection of participants, classification of interventions, deviations from intended interventions, missing data, measurement of outcomes, and selection of the reported result. Overall judgments reflect the highest level of concern across domains and the likely impact of bias on the study findings. Because all included studies were observational, residual confounding could not be fully excluded even in propensity-matched analyses. Time from index valve implantation to redo intervention was a key clinical confounder. Whether this variable was included in propensity matching was not clearly reported for Landes et al. (9) and was not available in the TriNetX-based Rosenzveig 2025 analysis; therefore, residual confounding by time-to-failure and failure mechanism remains possible.

### 3.3 Risk of bias assessment

Risk of bias was assessed using ROBINS-I for non-randomized studies, supplemented by the Newcastle-Ottawa Scale for cohort studies. The overall risk of bias varied across studies. Landes et al. (9) had the lowest risk of bias because it used an international registry-based propensity-matched design with standardized differences <0.1 across measured baseline covariates, although residual unmeasured confounding remained possible. Raschpichle et al. (11) was judged to have moderate risk of bias because it was retrospective and unmatched, with baseline imbalances between groups. Rosenzveig 2025 was also judged to have moderate risk of bias; although propensity-score matching was performed, outcomes were identified using ICD-10 and procedural codes from the TriNetX database, raising concerns regarding outcome misclassification and incomplete clinical detail.

Adjustment methods differed across the included studies. Landes et al. (9) used propensity-score matching across reported baseline clinical and procedural covariates, with standardized differences <0.1 after matching; however, the published report did not clearly indicate that time from index valve implantation to redo intervention was included as a matching variable. Similarly, Rosenzveig 2025 used propensity-score matching based on available TriNetX clinical covariates, but time from index procedure to valve failure was not available for assessment in the database-derived matched cohort. Therefore, although matching reduced measured baseline imbalance, it could not fully address confounding related to the timing and mechanism of valve failure, which remains central to the comparison between TAV-in-TAV and TAV-in-SAV.

Goppel et al. (22) was judged to have serious risk of bias because it was available only as a conference abstract and compared clinically different indications, with most TAV-in-TAV cases performed for intraoperative paravalvular regurgitation rather than established valve degeneration. Therefore, Goppel et al. (22) was excluded from the primary quantitative synthesis and retained only for narrative discussion. Overall, the risk-of-bias assessment supports cautious interpretation of the findings as exploratory and hypothesis-generating.

### 3.4 Outcomes

Meta-analysis was performed for four outcomes where at least two studies reported comparable data: 30-day mortality, 1-year mortality, mean transvalvular gradient at 30 days, and new permanent pacemaker implantation at 30 days. For binary outcomes, odds ratios (ORs) were calculated. For the continuous gradient outcome, mean differences (MDs) were calculated. Both common-effect and random-effects models were generated; the random-effects estimates are emphasized in the text because of the clinical heterogeneity and observational design of the included studies. Results are summarized in Table 6, and forest plots are shown in Figures 2–5. Given that each pooled analysis included only two studies, the pooled estimates should be interpreted cautiously. In this setting, heterogeneity statistics such as I^2^ are unstable and should not be considered strong evidence of true homogeneity. Because each pooled analysis was informed by only two studies, the quantitative results should be interpreted as exploratory and descriptive. With *k* = 2, random-effects estimates are unstable, between-study variance cannot be reliably estimated, and I^2^ values are not informative evidence of true homogeneity. Therefore, non-significant findings should not be interpreted as evidence of equivalence or absence of clinically meaningful differences.

**Table 6:** Meta-analysis summary.

Outcome	Studies pooled	Effect measure	Common-effect model	Random-effects model	I^2^	Interpretation
30-day mortality	2	OR	0.71 (95% CI 0.25–2.00); *p* = 0.519	0.71 (95% CI 0.25–2.00); *p* = 0.518	0.00%	No statistically significant difference; wide CI
1-year mortality	2	OR	1.00 (95% CI 0.47–2.09); *p* = 0.991	1.02 (95% CI 0.47–2.18); *p* = 0.967	0.00%	No statistically significant difference; wide CI
Mean gradient at 30 days	2	MD	−2.57 mmHg (95% CI −3.60 to −1.55); *p* < 0.0001	−2.71 mmHg (95% CI −4.07 to −1.34); *p* = 0.0001	25.10%	Lower gradient with TAV-in-TAV
New PPM at 30 days	2	OR	1.82 (95% CI 0.92–3.60); *p* = 0.086	1.85 (95% CI 0.94–3.65); *p* = 0.075	0.00%	Non-significant trend toward higher PPM with TAV-in-TAV

Summary of pooled estimates for outcomes included in the primary meta-analysis. Odds ratios were used for binary outcomes, and mean differences were used for continuous outcomes. Random-effects estimates were emphasized because of anticipated clinical and methodological heterogeneity among the included studies; common-effect estimates are shown as sensitivity analyses. Each pooled analysis included two full-text studies, and therefore heterogeneity statistics should be interpreted cautiously.

**Figure 2:**
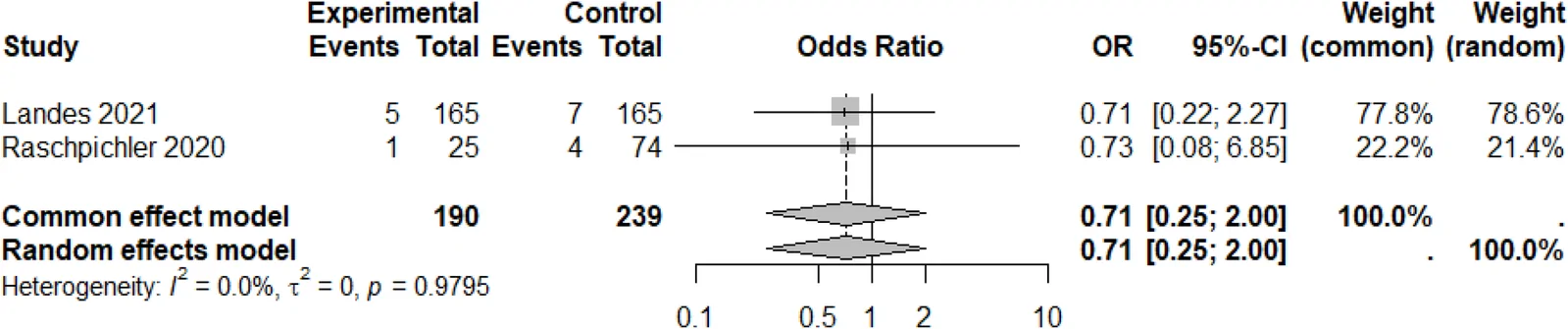
Forest plot of 30-day mortality (TAV-in-TAV vs. TAV-in-SAV).

**Figure 3:**
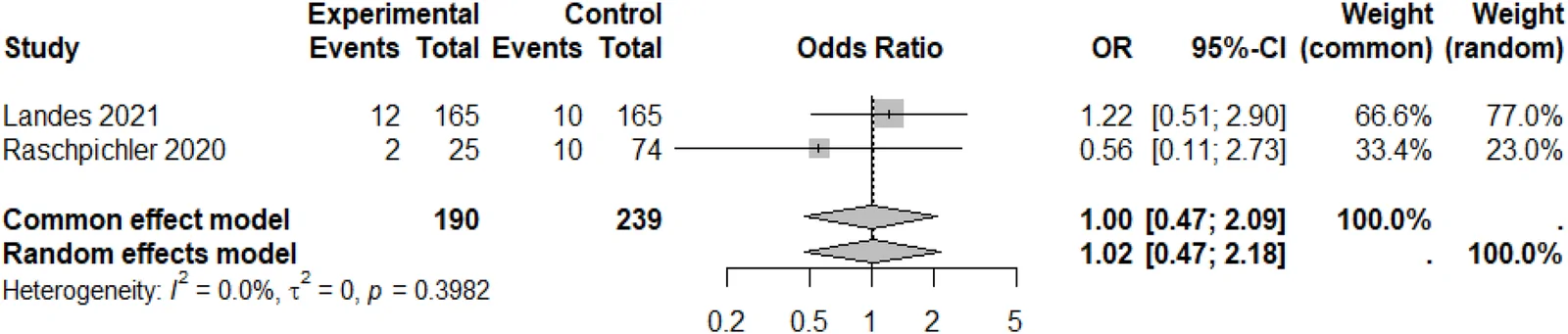
Forest plot of 1-year mortality (TAV-in-TAV vs. TAV-in-SAV).

**Figure 4:**
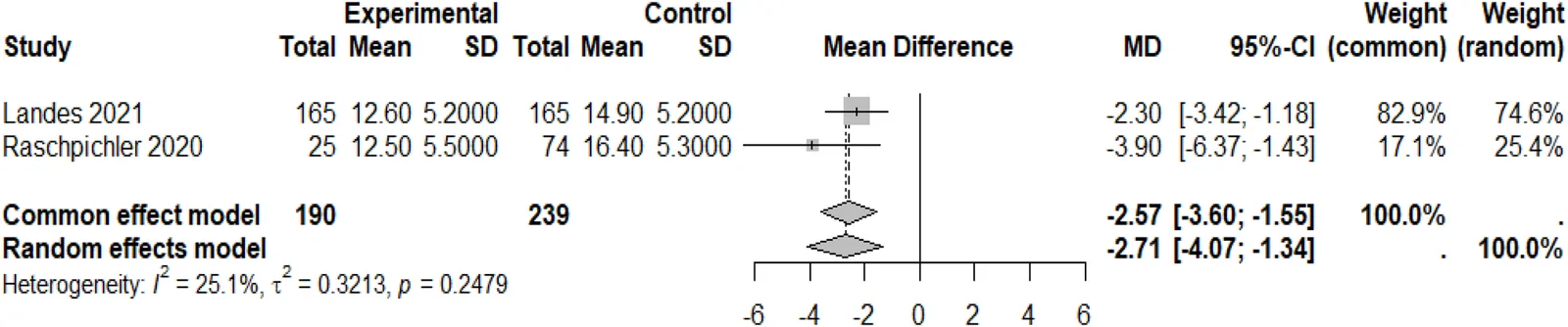
Forest plot of mean transvalvular gradient at 30 days (TAV-in-TAV vs. TAV-in-SAV).

**Figure 5:**
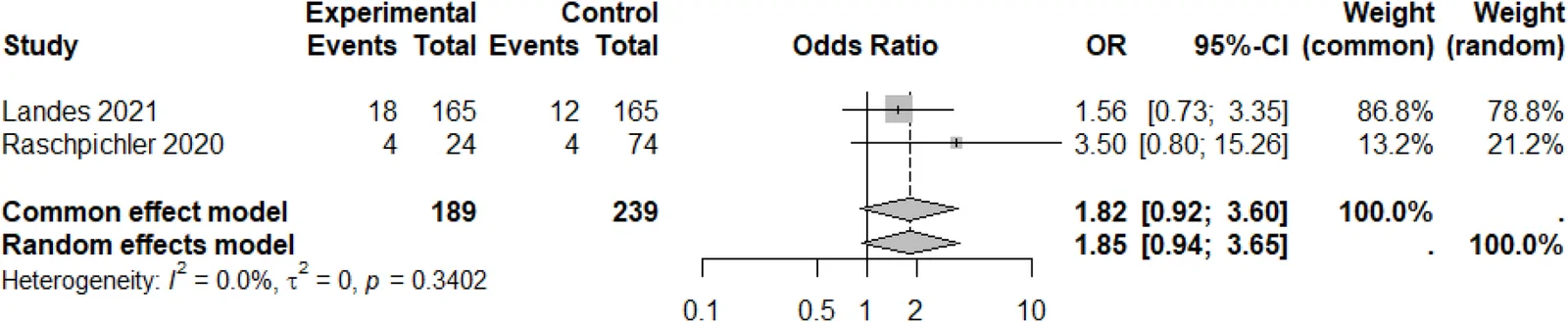
Forest plot of new permanent pacemaker implantation at 30 days (TAV-in-TAV vs. TAV-in-SAV).

**Figure 6:**
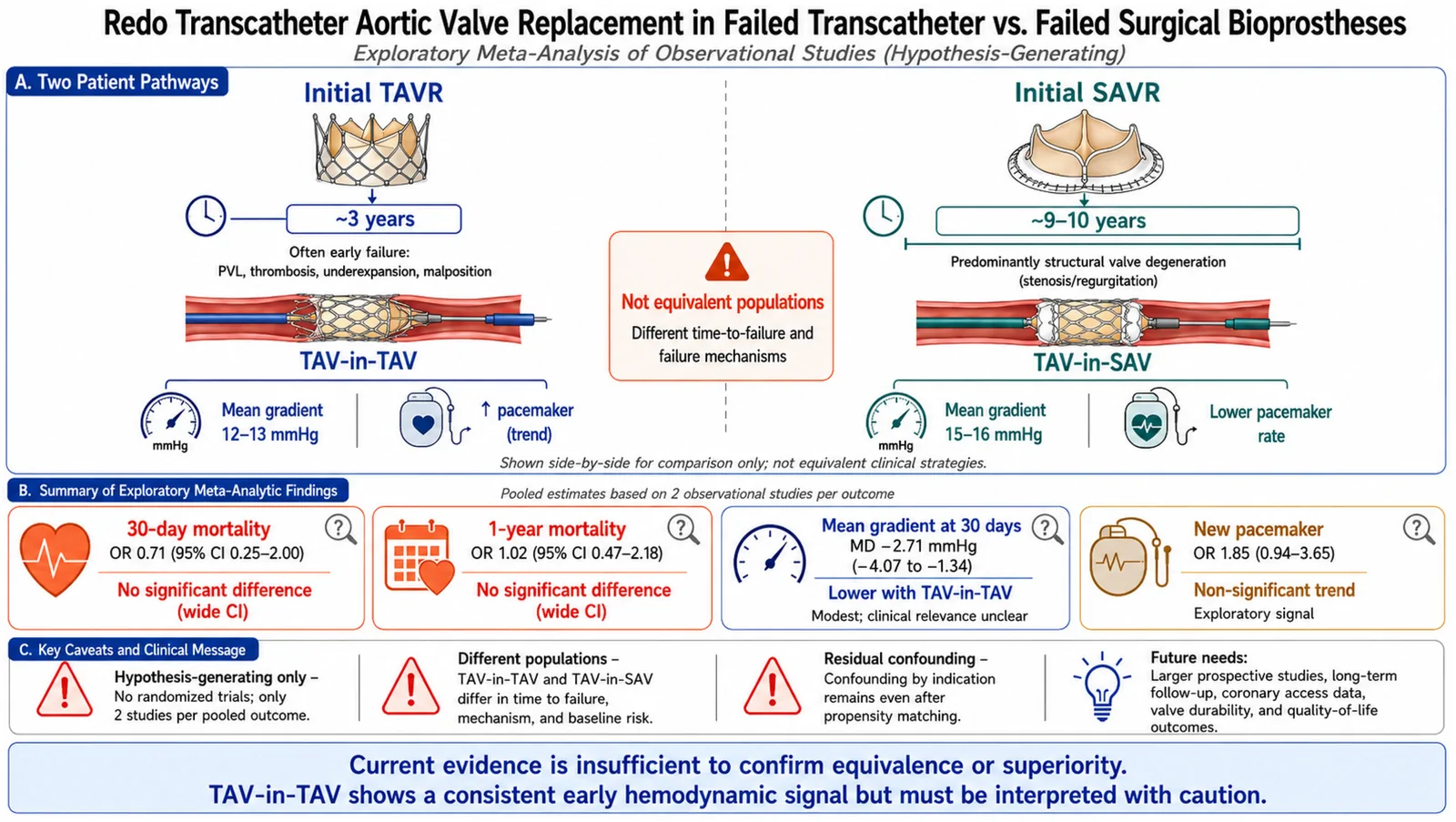
Central illustration emphasizing the non-equivalence of the two redo-TAVR populations. **(A)** Schematic representation of redo transcatheter aortic valve replacement after failed prior transcatheter valve implantation (TAV-in-TAV) versus redo transcatheter aortic valve replacement after failed surgical bioprosthesis (TAV-in-SAV). The two pathways are shown side-by-side for comparison only and should not be interpreted as equivalent clinical strategies. TAV-in-TAV generally occurred after a shorter interval from the index procedure and may reflect early valve dysfunction mechanisms such as paravalvular leak, thrombosis, underexpansion, or malposition, whereas TAV-in-SAV more often occurred after a longer interval and more commonly reflected structural valve degeneration. **(B)** Summary of exploratory pooled findings for 30-day mortality, 1-year mortality, 30-day mean transvalvular gradient, and new permanent pacemaker implantation. Estimates are based on two observational studies per pooled outcome and should be interpreted cautiously. **(C)** Key caveats and clinical implications, including the absence of randomized trials, limited number of studies, clinically different populations, residual confounding by indication, and the need for larger prospective studies with long-term follow-up, coronary access assessment, valve durability data, and quality-of-life outcomes. Overall, the current evidence is insufficient to confirm equivalence or superiority; TAV-in-TAV shows a consistent early haemodynamic signal but remains hypothesis-generating. CI, confidence interval; MD, mean difference; OR, odds ratio; PPM, permanent pacemaker; PVL, paravalvular leak; SAVR, surgical aortic valve replacement; TAV-in-SAV, transcatheter aortic valve replacement within a failed surgical bioprosthesis; TAV-in-TAV, transcatheter aortic valve replacement within a failed transcatheter valve; TAVR, transcatheter aortic valve replacement.

#### 3.4.1 30-day mortality

Two studies, Landes et al. (9) and Raschpichle et al. (11), contributed to the analysis of 30-day mortality. The pooled random-effects OR for 30-day mortality with TAV-in-TAV compared with TAV-in-SAV was 0.71 (95% CI 0.25–2.00; *p* = 0.518). The common-effect estimate was nearly identical: OR 0.71 (95% CI 0.25–2.00; *p* = 0.519). There was no observed statistical heterogeneity (I^2^ = 0.0%; *τ*^2^ = 0).

These findings do not demonstrate a statistically significant difference in 30-day mortality between TAV-in-TAV and TAV-in-SAV. However, the confidence interval is wide and remains compatible with clinically meaningful benefit or harm. Therefore, this result should be interpreted as the absence of clear evidence for a difference, not as evidence of equivalence or similar safety.

The individual study estimates were directionally consistent: Landes et al. (9) reported an OR of 0.71 (95% CI 0.22–2.27), while Raschpichle et al. (11) reported an OR of 0.73 (95% CI 0.08–6.85).

#### 3.4.2 1-year mortality

Two studies, Landes et al. (9) and Raschpichle et al. (11), reported 1-year mortality. The pooled random-effects OR was 1.02 (95% CI 0.47–2.18; *p* = 0.967), while the common-effect OR was 1.00 (95% CI 0.47–2.09; *p* = 0.991). No statistical heterogeneity was observed (I^2^ = 0.0%; *τ*^2^ = 0).

The pooled estimate was very close to the null, and the confidence interval crossed 1 widely. Thus, the analysis did not identify a statistically significant difference in 1-year mortality between TAV-in-TAV and TAV-in-SAV. Nevertheless, because only two observational studies contributed data and the confidence interval was broad, the available evidence is insufficient to exclude clinically meaningful differences in either direction.

Landes et al. (9) individually showed an OR of 1.22 (95% CI 0.51–2.90), whereas Raschpichle et al. (11) showed an OR of 0.56 (95% CI 0.11–2.73). Although the pooled heterogeneity estimate was 0%, the different directions of the individual point estimates reinforce the need for cautious interpretation.

#### 3.4.3 Mean transvalvular gradient at 30 days

Two studies, Landes et al. (9) and Raschpichle et al. (11), contributed data for the mean transvalvular gradient at 30 days. The pooled random-effects MD was −2.71 mmHg (95% CI −4.07 to −1.34; *p* = 0.0001), favoring TAV-in-TAV with lower postprocedural gradients. The common-effect estimate was similar but slightly smaller in magnitude: MD −2.57 mmHg (95% CI −3.60 to −1.55; *p* < 0.0001).

There was low-to-moderate statistical heterogeneity (I^2^ = 25.1%; *τ*^2^ = 0.3213), although this estimate should be interpreted cautiously because only two studies were included. Both studies showed lower mean gradients after TAV-in-TAV: Landes et al. (9) reported an MD of −2.30 mmHg (95% CI −3.42 to −1.18), and Raschpichle et al. (11) reported an MD of −3.90 mmHg (95% CI −6.37 to −1.43).

Overall, these results suggest a consistent signal toward lower early mean transvalvular gradients with TAV-in-TAV compared with TAV-in-SAV. However, the absolute magnitude of the difference was modest, approximately 2.5–3 mmHg, and its clinical relevance remains uncertain. The available data do not establish whether this early hemodynamic difference translates into improved survival, fewer heart failure events, better functional status, or reduced need for future reintervention.

This updated analysis is stronger than the previous gradient analysis because Goppel et al. (22) was removed from the primary analysis, reducing the concern related to abstract-only data, discharge rather than 30-day measurement, and different indications for TAV-in-TAV.

#### 3.4.4 New permanent pacemaker implantation at 30 days

Two studies, Landes et al. (9) and Raschpichle et al. (11), reported new permanent pacemaker implantation at 30 days. The pooled random-effects OR was 1.85 (95% CI 0.94–3.65; *p* = 0.075), while the common-effect OR was 1.82 (95% CI 0.92–3.60; *p* = 0.086). There was no observed heterogeneity (I^2^ = 0.0%; *τ*^2^ = 0).

Although the point estimate suggests a higher odds of new permanent pacemaker implantation after TAV-in-TAV, the confidence interval crosses 1, and the result did not reach conventional statistical significance. Therefore, this finding should be interpreted as an exploratory signal rather than definitive evidence of increased pacemaker risk.

The individual studies were directionally consistent but imprecise. Landes et al. (9) reported an OR of 1.56 (95% CI 0.73–3.35), while Raschpichle et al. (11) reported an OR of 3.50 (95% CI 0.80–15.26). The higher estimate in Raschpichle et al. (11) should be interpreted cautiously because of the small sample size and wide confidence interval.

Mechanistic explanations, such as differences in valve design, implantation depth, radial force, or interaction with the conduction system, remain plausible but cannot be confirmed from the current evidence.

## 4 Discussion

This systematic review identified four observational studies evaluating TAV-in-TAV versus TAV-in-SAV for failed aortic bioprostheses. However, only two full-text studies provided sufficiently comparable data for the primary quantitative analyses. The principal findings were: (1) no statistically significant difference in 30-day mortality between TAV-in-TAV and TAV-in-SAV; (2) no statistically significant difference in 1-year mortality; (3) a consistent early haemodynamic signal favouring TAV-in-TAV, with lower mean transvalvular gradients at 30 days; and (4) a non-significant trend toward higher new permanent pacemaker implantation after TAV-in-TAV. These findings should be interpreted as hypothesis-generating rather than confirmatory because the pooled analyses were based on only two observational studies per outcome, with wide confidence intervals for clinical endpoints and limited ability to exclude clinically meaningful benefit or harm.

The pooled analyses did not demonstrate a statistically significant difference in either 30-day or 1-year mortality. For 30-day mortality, the pooled random-effects OR was 0.71 (95% CI 0.25–2.00), while for 1-year mortality the pooled random-effects OR was 1.02 (95% CI 0.47–2.18). These estimates are imprecise and should not be interpreted as evidence of equivalence. Rather, they indicate that the currently available data are insufficient to detect or exclude clinically meaningful differences in mortality between TAV-in-TAV and TAV-in-SAV. This is particularly important because the analyses were based on only two studies and a small number of events. The findings are broadly consistent with Landes et al., the largest included study, which reported no significant difference in 30-day or 1-year mortality in propensity-matched cohorts. However, the available evidence remains underpowered and observational.

A key consideration is that TAV-in-TAV and TAV-in-SAV may not represent fully comparable clinical entities. Across the included studies, the time from index valve implantation to redo intervention was consistently shorter for TAV-in-TAV, approximately 3 years, compared with approximately 9–10 years for TAV-in-SAV. Early failure after TAVR may reflect mechanisms such as leaflet thrombosis, valve underexpansion, malposition, early paravalvular regurgitation, or device-related dysfunction, whereas late failure after SAVR more often reflects progressive structural valve degeneration. Therefore, this review should be interpreted as a comparison of outcomes after redo TAVR in two different prior-valve populations rather than as a definitive comparison of two equivalent reintervention strategies. Residual confounding by indication and failure mechanism is likely, even in propensity-matched cohorts.

TAV-in-TAV was associated with lower mean transvalvular gradients at 30 days. In the primary analysis excluding Goppel et al. (22), the pooled random-effects MD was −2.71 mmHg (95% CI −4.07 to −1.34), favouring TAV-in-TAV. Both Landes et al. (9) and Raschpichle et al. (11) showed the same direction of effect, with lower gradients after TAV-in-TAV. Heterogeneity was low to moderate (I^2^ = 25.1%), although this estimate is unstable because only two studies were included. Compared with the previous analysis,s including Goppel, removal of the abstract-only study reduced methodological and clinical heterogeneity and provided a more appropriate estimate based on 30-day data.

The clinical relevance of this gradient difference remains uncertain. Although lower residual gradients may suggest better early haemodynamic performance, the absolute difference was modest, approximately 2.5–3 mmHg. The available evidence does not establish whether this early difference translates into improved survival, fewer heart failure events, better functional status, reduced reintervention, or improved long-term valve durability. Therefore, the gradient result should be framed as a consistent early haemodynamic signal rather than definitive evidence of clinically superior outcomes.

TAV-in-TAV showed a non-significant trend toward higher new permanent pacemaker implantation. The pooled random-effects OR was 1.85 (95% CI 0.94–3.65; *p* = 0.075), while the common-effect estimate was similar. Although the point estimate suggests increased odds of new pacemaker implantation after TAV-in-TAV, the confidence interval crossed the null,l and the result did not reach conventional statistical significance. This should therefore be interpreted as an exploratory signal rather than definitive evidence of increased conduction risk.

Potential mechanisms may include differences in valve design, implantation depth, radial force, and interaction between the redo transcatheter valve and the prior transcatheter frame. However, the current evidence is insufficient to support a causal mechanistic conclusion. Larger studies with detailed procedural data, valve type, implantation depth, baseline conduction status, and standardized pacemaker indications are needed to clarify this possible association.

The largest and most methodologically robust included study was the Redo-TAVR registry by Landes et al. (9), which used propensity-score matching and reported no significant difference in 30-day mortality, 1-year mortality, or procedural safety between TAV-in-TAV and TAV-in-SAV. Landes also reported lower 30-day mean gradients after TAV-in-TAV and higher procedural success, largely driven by fewer residual high gradients. Raschpichler et al. similarly reported lower 30-day gradients and no significant difference in mortality, although the study was smaller and unmatched. Together, these studies provide a consistent signal of favorable early hemodynamics after TAV-in-TAV, but they remain insufficient to establish comparative clinical superiority or long-term safety.

When compared with the alternative of redo surgical aortic valve replacement, a 2025 meta-analysis by Dimitriadis et al. (20) found that valve-in-valve TAVR, predominantly TAV-in-SAV, was associated with lower 30-day and 1-year mortality and fewer bleeding events than redo surgery but with higher mean gradients and paravalvular leak. This context supports the role of transcatheter valve-in-valve therapy as a less invasive alternative to redo surgery in selected patients. However, these findings should not be directly extrapolated to establish the comparative safety of TAV-in-TAV, because redo TAVR after prior TAVR has unique anatomical and procedural considerations.

Longer-term evidence remains very limited. Only Landes and Raschpichler contributed to the pooled 1-year mortality analysis, while Rosenzveig et al. (10) provided 3-year outcomes using the TriNetX database. Rosenzveig reported no statistically significant difference in 3-year mortality, MACE, stroke, major bleeding, MAKE, or endocarditis between matched TAV-in-TAV and TAV-in-SAV cohorts. However, this study relied on ICD-10 and procedural codes, which may introduce outcome misclassification and residual confounding because detailed clinical, echocardiographic, procedural, and valve-specific data were not available. Therefore, the 3-year data are useful but should be interpreted cautiously. Longer follow-up with adjudicated clinical endpoints and core laboratory echocardiography is required to determine whether early hemodynamic differences persist and whether they affect long-term outcomes.

Future coronary access is an important issue that was not adequately captured in the included studies. This is particularly relevant for TAV-in-TAV because redoing TAVR may create overlapping valve frames, higher neoskirt formation, commissural misalignment, leaflet displacement, and potential sinus sequestration. These anatomical changes may make future coronary angiography or percutaneous coronary intervention more difficult, especially in younger patients who may require decades of cardiac care. Therefore, procedural planning for TAV-in-TAV should not be based only on short-term mortality or early gradients but should also consider coronary height, sinus dimensions, valve design, commissural alignment, and the feasibility of future coronary access or additional valve procedures.

### 4.1 Strengths & limitations

This review has several strengths. It addresses an important and emerging clinical question as TAVR expands to younger and lower-risk patients who may outlive their first bioprosthetic valve and require future reintervention. The search was comprehensive across five major databases, predefined eligibility criteria were applied, and risk of bias was assessed using validated tools. In addition, this review provides a structured comparison of baseline characteristics, procedural details, haemodynamic outcomes, and clinical safety outcomes across the available comparative studies. By separating studies suitable for primary quantitative synthesis from those suitable only for narrative discussion, particularly Goppel et al. (22), the revised analysis provides a more cautious and transparent summary of the current evidence.

Several limitations should be acknowledged. First, all included studies were observational, and no randomized trials were available. Therefore, selection bias, residual confounding, and confounding by indication remain major concerns. Second, TAV-in-TAV and TAV-in-SAV patients may represent clinically distinct populations with different index procedures, different times from index valve implantation to failure, and different mechanisms of valve dysfunction. This is central to the interpretation of the findings because TAV-in-TAV more often occurred after a shorter interval from the index procedure, whereas TAV-in-SAV generally occurred after a longer interval and more often reflected structural valve degeneration.

Third, only two studies contributed to each primary pooled outcome, making the meta-analytic estimates statistically fragile. In this context, random-effects estimates are unstable, between-study variance cannot be estimated reliably, and I^2^ values or tests of heterogeneity should not be overinterpreted. The pooled estimates should therefore be viewed as exploratory and hypothesis-generating rather than confirmatory.

Fourth, formal assessment of publication bias using funnel plots or regression-based tests was not possible because fewer than 10 studies were included. Publication bias and selective outcome reporting remain plausible, particularly because small observational redo-TAVR cohorts may preferentially report favorable early haemodynamic outcomes while longer-term clinical endpoints are less consistently reported. Therefore, the possibility that the available evidence overrepresents positive early haemodynamic findings cannot be excluded.

Fifth, Goppel et al. (22) was available only as a conference abstract and included a TAV-in-TAV cohort predominantly treated for intraoperative paravalvular regurgitation rather than established valve degeneration. Because this population differed substantially from the target redo-valve-failure population, the study was excluded from the primary quantitative synthesis and discussed narratively only. Sixth, Rosenzveig 2025 used ICD-10 and procedural codes from the TriNetX database, which may introduce outcome misclassification and does not allow detailed adjustment for valve type, operator experience, anatomical features, failure mechanism, or time from index procedure to valve failure.

Seventh, some continuous outcomes required conversion from median/IQR to mean/SD, which may introduce approximation error. Finally, follow-up was limited, and several clinically important outcomes were poorly reported or absent, including future coronary access, valve durability, quality of life, functional status, and the need for subsequent reintervention. Larger prospective studies with adjudicated outcomes, detailed anatomical and procedural data, and longer follow-up are required to clarify the comparative role of TAV-in-TAV and TAV-in-SAV within lifetime valve-management strategies.

### 4.2 Clinical implications

The clinical importance of this comparison lies in the fact that TAV-in-TAV and TAV-in-SAV are increasingly encountered as competing redo-TAVR scenarios, but they arise from different index procedures, different time horizons, and different mechanisms of valve failure. For clinicians planning an index valve strategy, the distinction is important because the consequences of choosing TAVR versus SAVR may extend beyond the first procedure and influence the feasibility, risks, and haemodynamic profile of future reintervention. In this review, TAV-in-TAV was associated with lower early mean gradients, but this signal occurred in a population with substantially shorter time to redo intervention and potentially different failure mechanisms. Therefore, the finding should not be interpreted as evidence that TAV-in-TAV is clinically superior to TAV-in-SAV. From a practical standpoint, the current evidence supports careful individualized planning rather than a single preferred redo strategy. In patients with failed prior TAVR, early haemodynamic performance after TAV-in-TAV may be acceptable, but the possible trade-offs include uncertain durability, a non-significant signal toward higher pacemaker implantation, and potentially more complex future coronary access because of overlapping valve frames and neoskirt formation. In patients with failed surgical bioprostheses, TAV-in-SAV remains a more established valve-in-valve scenario, but residual gradients and patient-prosthesis mismatch remain important concerns, particularly in small surgical valves. Thus, this comparison matters clinically because it informs lifetime valve-management decisions, patient counseling, index valve selection, and redo-procedure planning, while also showing that current evidence is insufficient to claim equivalence or superiority between the two redo-TAVR populations.

## 5 Conclusion

In this systematic review with exploratory meta-analysis of limited observational evidence, TAV-in-TAV and TAV-in-SAV were not associated with statistically significant differences in 30-day or 1-year mortality. However, the confidence intervals were wide, and clinically meaningful differences cannot be excluded. TAV-in-TAV was associated with lower early mean transvalvular gradients, but the magnitude of this difference was modest, and its long-term clinical relevance remains uncertain. A non-significant trend toward higher permanent pacemaker implantation after TAV-in-TAV was observed and should be considered hypothesis-generating. Because the available evidence is sparse, observational, and clinically heterogeneous, these findings should not be interpreted as proof of equivalence or definitive comparative safety. Larger prospective studies with adjudicated outcomes, detailed anatomical and procedural data, and longer follow-up are needed to clarify the role of TAV-in-TAV within lifetime valve management strategies.

## Data Availability

The original contributions presented in the study are included in the article/Supplementary Material, further inquiries can be directed to the corresponding author.
