# Supplementary material for: Transcatheter aortic valve replacement in failed transcatheter versus failed surgical bioprostheses: a systematic review with exploratory meta-analysis
Source: Front Cardiovasc Med. 2026 Sep 18;13:1870804. doi: 10.3389/fcvm.2026.1870804 (PMC13630731; doi:10.3389/fcvm.2026.1870804)
Supplement: Supplementary file 1 [file Table1.docx]

# 1. Search and Screening

*Appendix Table A1. Detailed Search Strategy Across Databases*

| **Database** | **Search strategy** |
| --- | --- |
| **PubMed/MEDLINE** | (("Transcatheter Aortic Valve Replacement"[Mesh] OR "transcatheter aortic valve replacement"[tiab] OR "transcatheter aortic valve implantation"[tiab] OR TAVR[tiab] OR TAVI[tiab]) AND ("valve-in-valve"[tiab] OR "valve in valve"[tiab] OR ViV[tiab] OR "redo TAVR"[tiab] OR "repeat TAVR"[tiab] OR "reintervention"[tiab] OR "redo transcatheter aortic valve replacement"[tiab]) AND ("failed transcatheter"[tiab] OR "failed TAVR"[tiab] OR "failed TAVI"[tiab] OR "degenerated TAVR"[tiab] OR "degenerated TAVI"[tiab] OR "transcatheter valve failure"[tiab] OR "TAV-in-TAV"[tiab] OR "TAVR-in-TAVR"[tiab] OR "TAVI-in-TAVI"[tiab] OR ViTAVR[tiab] OR "valve-in-TAVR"[tiab]) AND ("failed surgical"[tiab] OR "failed surgical bioprosthesis"[tiab] OR "degenerated surgical bioprosthesis"[tiab] OR "surgical bioprosthetic valve failure"[tiab] OR "failed SAVR"[tiab] OR "surgical aortic valve"[tiab] OR SAVR[tiab] OR "TAV-in-SAV"[tiab] OR "TAVR-in-SAVR"[tiab] OR "TAVI-in-SAVR"[tiab] OR ViSAVR[tiab] OR "valve-in-SAVR"[tiab])) |
| **Embase** | ('transcatheter aortic valve implantation'/exp OR 'transcatheter aortic valve replacement':ti,ab OR 'transcatheter aortic valve implantation':ti,ab OR tavr:ti,ab OR tavi:ti,ab) AND ('valve in valve':ti,ab OR 'valve-in-valve':ti,ab OR viv:ti,ab OR 'redo tavr':ti,ab OR 'repeat tavr':ti,ab OR 'redo transcatheter aortic valve replacement':ti,ab OR reintervention:ti,ab) AND ('failed transcatheter':ti,ab OR 'failed tavr':ti,ab OR 'failed tavi':ti,ab OR 'degenerated tavr':ti,ab OR 'degenerated tavi':ti,ab OR 'transcatheter valve failure':ti,ab OR 'tav-in-tav':ti,ab OR 'tavr-in-tavr':ti,ab OR 'tavi-in-tavi':ti,ab OR vitavr:ti,ab OR 'valve-in-tavr':ti,ab) AND ('failed surgical':ti,ab OR 'failed surgical bioprosthesis':ti,ab OR 'degenerated surgical bioprosthesis':ti,ab OR 'surgical bioprosthetic valve failure':ti,ab OR 'failed savr':ti,ab OR 'surgical aortic valve':ti,ab OR savr:ti,ab OR 'tav-in-sav':ti,ab OR 'tavr-in-savr':ti,ab OR 'tavi-in-savr':ti,ab OR visavr:ti,ab OR 'valve-in-savr':ti,ab) |
| **Scopus** | TITLE-ABS-KEY(("transcatheter aortic valve replacement" OR "transcatheter aortic valve implantation" OR TAVR OR TAVI) AND ("valve-in-valve" OR "valve in valve" OR ViV OR "redo TAVR" OR "repeat TAVR" OR "redo transcatheter aortic valve replacement" OR reintervention) AND ("failed transcatheter" OR "failed TAVR" OR "failed TAVI" OR "degenerated TAVR" OR "degenerated TAVI" OR "transcatheter valve failure" OR "TAV-in-TAV" OR "TAVR-in-TAVR" OR "TAVI-in-TAVI" OR ViTAVR OR "valve-in-TAVR") AND ("failed surgical" OR "failed surgical bioprosthesis" OR "degenerated surgical bioprosthesis" OR "surgical bioprosthetic valve failure" OR "failed SAVR" OR "surgical aortic valve" OR SAVR OR "TAV-in-SAV" OR "TAVR-in-SAVR" OR "TAVI-in-SAVR" OR ViSAVR OR "valve-in-SAVR")) |
| **Web of Science Core Collection** | TS=(("transcatheter aortic valve replacement" OR "transcatheter aortic valve implantation" OR TAVR OR TAVI) AND ("valve-in-valve" OR "valve in valve" OR ViV OR "redo TAVR" OR "repeat TAVR" OR "redo transcatheter aortic valve replacement" OR reintervention) AND ("failed transcatheter" OR "failed TAVR" OR "failed TAVI" OR "degenerated TAVR" OR "degenerated TAVI" OR "transcatheter valve failure" OR "TAV-in-TAV" OR "TAVR-in-TAVR" OR "TAVI-in-TAVI" OR ViTAVR OR "valve-in-TAVR") AND ("failed surgical" OR "failed surgical bioprosthesis" OR "degenerated surgical bioprosthesis" OR "surgical bioprosthetic valve failure" OR "failed SAVR" OR "surgical aortic valve" OR SAVR OR "TAV-in-SAV" OR "TAVR-in-SAVR" OR "TAVI-in-SAVR" OR ViSAVR OR "valve-in-SAVR")) |
| **Cochrane CENTRAL** | ("transcatheter aortic valve replacement" OR "transcatheter aortic valve implantation" OR TAVR OR TAVI) AND ("valve-in-valve" OR "valve in valve" OR ViV OR "redo TAVR" OR "repeat TAVR" OR "redo transcatheter aortic valve replacement" OR reintervention) AND ("failed transcatheter" OR "failed TAVR" OR "failed TAVI" OR "degenerated TAVR" OR "degenerated TAVI" OR "transcatheter valve failure" OR "TAV-in-TAV" OR "TAVR-in-TAVR" OR "TAVI-in-TAVI" OR ViTAVR OR "valve-in-TAVR") AND ("failed surgical" OR "failed surgical bioprosthesis" OR "degenerated surgical bioprosthesis" OR "surgical bioprosthetic valve failure" OR "failed SAVR" OR "surgical aortic valve" OR SAVR OR "TAV-in-SAV" OR "TAVR-in-SAVR" OR "TAVI-in-SAVR" OR ViSAVR OR "valve-in-SAVR") |
